# The OB-GYN Take on GPT: Objective Assessment of Artificial Intelligence Models in Patient Education

**DOI:** 10.7759/cureus.101600

**Published:** 2026-01-15

**Authors:** Lindsey Burleson, Ella Boardley, Alexandra LaShell, Anthony Shanks

**Affiliations:** 1 Obstetrics and Gynecology, Indiana University School of Medicine, Indianapolis, USA

**Keywords:** artifical intelligence, health education & awareness, large language model, obstetrics & gynecology, patient education

## Abstract

Large language models (LLMs), a subset of generative artificial intelligence, have garnered significant attention in healthcare. While existing literature has examined the role of LLMs in medical education, primarily focusing on their ability to answer examination questions, few studies have investigated their potential in patient education and assessed the role of LLMs in medical education, few studies evaluate their role in patient education, and none have specifically evaluated their use in OB-GYN. Using a standardized rubric, we assessed the responses of three LLMs and the American College of Obstetricians and Gynecologists (ACOG) on 10 clinical questions that reflect common patient concerns in OB-GYN, which broadly represent common patient queries in OB-GYN. CoPilot by Microsoft demonstrated higher scores by expert reviewers; however, evaluators were more likely to recommend ACOG frequently asked questions (FAQs) or ChatGPT to patients when providing clinical counseling. This study supports the assertion that LLMs have a role in patient education and counseling in OB-GYN.

## Introduction

Since the creation of the internet, patients have had unprecedented access to medical information. However, the way patients seek medical knowledge is evolving. Traditional web searches are being replaced by interactions with large language models (LLMs) like ChatGPT [1]. This broader trend in digital engagement reflects a growing reliance on artificial intelligence (AI) tools by patients to understand symptoms, explore treatment options, and make informed decisions about their care. 

LLMs are a subset of AI that generate responses based on patterns learned from large text datasets [2]. Their conversational nature makes them appealing for answering medical questions and may augment patient education by making information more accessible and understandable [3]. 

Though convenient, these models can generate information that is incomplete or misleading, posing risks when used without professional oversight. Data on LLM accuracy and relevance to OB-GYN is limited, and there are no standard guidelines for evaluating their performance in healthcare [4]. In addition, previous studies on LLM evaluation have focused on clinician-centered questions rather than looking at patient-facing materials [5]. As LLMs become more integrated into healthcare, clear evaluation frameworks and physician oversight will be critical to ensure safe and accurate patient guidance. 

The objectives of this study are to evaluate the accuracy, quality, and appropriateness of medical responses generated by LLMs and to compare them with expert-reviewed frequently asked questions (FAQs) provided by the American College of Obstetricians and Gynecologists (ACOG). Using a structured evaluation framework, expert physicians will assess LLM-generated content against objective clinical standards to determine its suitability for patient use. This analysis aims to inform best practices for the safe integration of LLMs into patient education while maintaining the standards of evidence-based medicine. 

## Materials and methods

Study design

Ten topics representative of the specialty were selected from ACOG FAQs (Table 1). ACOG provides a wealth of resources for patients, and their FAQs are created by experts and contain easy-to-understand information on a wide range of topics in Women’s Health. These FAQs provided the questions that were used to be evaluated by LLMs. One clinical question per topic was entered into three publicly accessible LLMs on April 2, 2023: ChatGPT 3.5 (OpenAI), CoPilot (Microsoft), and Gemini (Google). This was done using a zero-shot technique. Zero-shot means the model can answer a question or perform a task without seeing any example prompts or demonstrations of that task in the current interaction. 

**Table 1:** List of OB-GYN Topics and Representative Questions for the LLM LLM, large language model.

Topic	Clinical Question
Vaginal Birth After Cesarean (VBAC)	What are the risks of VBAC?
Emergency Contraception	How quickly after unprotected sex do I have to use emergency contraception?
Hysterectomy	What should I expect after a hysterectomy?
Mammogram	When should I start having screening mammography?
Cervical Cancer	What is cervical cancer screening?
HPV vaccination	Is the HPV vaccine safe and effective?
Breastfeeding	How does breastfeeding benefit me?
Osteoporosis	What are some risk factors for osteoporosis?
Abortion Safety	Is abortion safe?
Intimate Partner Violence	What is intimate partner violence?

The first response from each LLM was collected. Subject matter experts from the Department of OB-GYN at Indiana University School of Medicine evaluated deidentified LLM responses and included the blinded corresponding ACOG FAQs. 

Study population/sample size 

Five experts reviewed outputs from each of the 10 questions across three LLMs and ACOG FAQs, scoring 40 distinct items (10 items from each source). 

Study measures**

Experts then rated each response output (Likert scale 1, lowest; 5, highest) for clinical accuracy, specificity, absence of confabulations (incorrect details) [6], and whether references were provided (Likert scale 1, lowest; 5, highest). Scores for each LLM and ACOG FAQ were averaged by category. Reviewers also selected their preferred response for patient counseling. 

Ethics statement 

The study was deemed to be IRB exempt (Indiana University School of Medicine #22463). 

Statistical analysis 

Descriptive statistics with one-way analysis of variance (ANOVA) and Tukey’s HSD (honestly significant difference), when appropriate, were performed using SPSS 29 (IBM Corp., Armonk, NY). Data is presented as mean for each parameter with statistical significance defined as p <0.05. 

## Results

Among the LLMs evaluated, ACOG AI, ChatGPT, Gemini, and CoPilot, CoPilot scored the highest in clinical accuracy, specificity, absence of confabulations, and quality of references. While there was no statistically significant difference in conciseness across models, reviewer preferences varied. 

CoPilot scored the highest in clinical accuracy (mean 4.52, SD 0.71) (Table 2) with a mean difference that was statistically significant (F (3, 196) = (3.19), p = 0.025). The mean value of clinical accuracy was statistically different between ACOG and CoPilot (p = 0.02, 95% CI = (-0.87, -0.49)). 

**Table 2:** Comparison of LLMs on Clinical Questions Parameters evaluated on Likert scale of 1-5 (max 5). Means are reported in the table. ANOVA is used to compare variables between LLMs as well as Tukey’s HSD test. p-Value <0.05 is considered significant. ACOG, American College of Obstetricians and Gynecologists; LLM, large language model; ANOVA, analysis of variance; HSD, honestly significant difference.

Domain	ACOG (Mean ± SD)	ChatGPT (Mean ± SD)	CoPilot (Mean ± SD)	Gemini (Mean ± SD)	F (df)	p-Value
Clinical Accuracy	4.06 ± 1.02	4.42 ± 0.7	4.52 ± 0.71	4.4 ± 0.7	3.16 (3, 196)	0.03
Specificity	3.48 ± 1.31	3.98 ± 1.0	4.46 ± 0.79	4.22 ± 0.86	8.57 (3, 196)	<0.01
Conciseness	4.16 ± 0.91	4.22 ± 0.79	3.96 ± 1.12	4.24 ± 0.94	0.91 (3, 196)	0.44
Confabulation	3.94 ± 1.0	4.04 ± 1.03	4.48 ± 0.68	4.2 ± 0.97	3.22 (3, 196)	0.03
References	1.62 ± 0.92	1.66 ± 1.04	4.78 ± 0.46	3.82 ± 1.56	109.45 (3, 196)	<0.01

CoPilot scored the highest in specificity (mean 4.46, SD 0.99). One-way ANOVA revealed that there was a statistically significant difference in means for clinical accuracy between at least two groups (F (3, 196) = (3.19), p < 0.01). Tukey’s HSD test for multiple comparisons found that the mean value of specificity was statistically different between ACOG and CoPilot (p < 0.01, 95% CI = (-1.50, -0.46)) and Gemini (p < 0.01, 95% CI = (-1.26, -0.22)). 

There was no statistically significant difference in conciseness between the LLMs (F (3, 196) = (0.91), p = 0.44). Post hoc analysis was not conducted for conciseness, as ANOVA did not detect a statistically significant difference between LLMs for this domain. 

CoPilot scored the highest in the absence of confabulations (mean 4.48, SD 0.68) (Table 2). One-way ANOVA revealed that there was a statistically significant difference in means for the absence of confabulations between at least two groups (F (3, 196) = (3.22), p = 0.02). Tukey’s HSD test for multiple comparisons found that the mean value of the absence of confabulations was statistically different between ACOG and CoPilot (p = 0.02, 95% CI = (-1.02, -0.0.06)) (Table 3). 

**Table 3:** Comparative Analysis of LLM Performance* *Post-hoc Tukey HSD results with 95% confidence intervals. LLM, large language model; HSD, honestly significant difference.

Domain	Comparison	p-Value	95% CI (Lower, Upper)
Clinical Accuracy	ACOG vs ChatGPT	0.109	(-0.77, 0.051)
Clinical Accuracy	ACOG vs CoPilot	0.02	(-0.87, -0.49)
Clinical Accuracy	ACOG vs Gemini	0.144	(-0.75, 0.071)
Clinical Accuracy	ChatGPT vs CoPilot	0.922	(-0.51, 0.31)
Clinical Accuracy	ChatGPT vs Gemini	0.999	(-0.39, 0.43)
Clinical Accuracy	CoPilot vs Gemini	0.874	(-0.29, 0.53)
Specificity	ACOG vs ChatGPT	0.068	(-1.02, 0.024)
Specificity	ACOG vs CoPilot	<0.01	(-1.26, -0.22)
Specificity	ACOG vs Gemini	0.002	(-1.26, -0.22)
Specificity	ChatGPT vs CoPilot	0.086	(-1.0041, 0.0041)
Specificity	ChatGPT vs Gemini	0.636	(-0.76, 0.28)
Specificity	CoPilot vs Gemini	0.636	(-0.28, 0.76)
Confabulation	ACOG vs ChatGPT	0.950	(-0.58, -0.38)
Confabulation	ACOG vs CoPilot	0.02	(-1.02, 0.06)
Confabulation	ACOG vs Gemini	0.501	(-0.74, 0.22)
Confabulation	ChatGPT vs CoPilot	0.087	(-0.92, 0.042)
Confabulation	ChatGPT vs Gemini	0.825	(-0.64, 0.32)
Confabulation	CoPilot vs Gemini	0.435	(-0.20, 0.76)
References	ACOG vs ChatGPT	0.998	(-0.60, 0.52)
References	ACOG vs CoPilot	<0.01	(-3.72, -2.6)
References	ACOG vs Gemini	<0.01	(-2.76, -1.64)
References	ChatGPT vs CoPilot	<0.01	(-3.68, -2.56)
References	ChatGPT vs Gemini	<0.01	(-2.72, -1.06)
References	CoPilot vs Gemini	<0.001	(0.41, 1.52)

CoPilot scored the highest in references (mean 4.78, SD 0.46) (Table 2). A one-way ANOVA revealed a statistically significant difference in the mean number of references between at least two groups, F(3, 196) = 109.45, p < 0.01. Follow-up analysis using Tukey’s HSD test showed that CoPilot had a significantly higher mean number of references compared to all other LLMs. The largest difference was observed between CoPilot and Gemini (p < 0.01, 95% CI (0.40, 1.51)) (Table 3). 

CoPilot was the highest overall scoring LLM (mean 22.20, SD 2.57) (Table 2), and this was statistically significant (F (3, 196) = 24.22, p < 0.01). Raters had good reliability with an intraclass coefficient of 0.85 (95% CI (0.77, 0.91)). Reviewers also selected their preferred answer for each clinical question from four blinded responses. ACOG (14) and ChatGPT (14) were most preferred, while CoPilot (10) was least preferred despite its strong objective performance. 

## Discussion

The need for critical assessment of AI platforms is imperative to patient safety and the patient-physician relationship [7]. Limited data is available to assess the accuracy of LLMs for patient-level advice, and topics in OB-GYN have not been evaluated in this matter. Clinicians in our study objectively rated Co-Pilot the highest on four of five objective metrics, such as accuracy and providing references. This was a similar finding to where Co-Pilot received the highest scores for reliability, quality, and overall scores for patient education materials related to chemotherapy [8]. However, ACOG and ChatGPT were the preferred LLMs. The latter platforms may be favored because of the familiarity of the output or the conversational nature of the answers provided. This was found to be similar in other studies as well, where ChatGPT was scored highest for helpfulness and understandability [7,8]. 

Strengths of this study include the breadth of topics covered, blinding of the responses by the LLMs, and the use of an objective rubric for evaluation with multiple experts. Limitations include our sample size of experts and the questions chosen, which may introduce selection bias. Additionally, this study used responses from one search at a single point in time. Since LLMs are always changing and improving, the generalizability of this study is limited. Furthermore, there was a lack of interaction with the LLM following the initial prompt, and users may have had a different preference when given the opportunity to ask follow-up questions. The conversational capacity of LLMs is one of the advantages, and why patients may be attracted to these new technologies. It would also be important to note if LLM responses remain accurate and do not accumulate errors [5]. As highlighted in a recent analysis, maximizing the benefits of AI in clinical settings requires addressing challenges related to data quality, algorithmic bias, and the continuing need for human oversight [9].

Other studies have similarly found that patients seeking a comprehensive understanding of their conditions could benefit from LLM for education. However, concerns about readability may hinder accessibility for certain patient groups [10]. In a study that compared ChatGPT to specialist doctors on answering questions about chronic disease, ChatGPT delivered appropriate and easily understandable responses to questions about diagnoses and treatment options. However, it tends to be less effective in explaining diagnostic tests and offering guidance on complex management strategies [11]. Previous studies have been carried out in high-income countries, which raises concerns about their applicability to low- and middle-income settings that have distinct healthcare needs and infrastructure [10]. 

In future studies, it could be important to look at how the type of question asked to LLMs affects the results. One study compared Google Search with ChatGPT responses to medical questions written from a patient perspective. They found that ChatGPT did better when offering general medical knowledge, but Google Search did better at providing medical recommendations [12]. Prompts entered into LLMs can affect the quality of the information provided. 

As AI accelerates the pace of innovation in healthcare, it is imperative that objective measures of assessment are used to evaluate its impact. With time, LLMs will likely improve on these different constructs that we evaluated. It is incumbent on the user of these LLMs to factor in these considerations when evaluating the output. Our study shows that LLMs can be a valuable resource for patient education. Future research is needed on the optimal method to query responses in LLMs and patient preferences. 

## Conclusions

Patients with health-related questions increasingly search online, making accurate information integral to medical management. While this behavior improves access, it exposes patients to harmful misinformation. As AI continues to drive rapid innovation in healthcare, it is essential to use objective evaluation methods to assess its impact.

Our study highlights the potential of LLMs to provide accessible health information for patients. The findings suggest that LLMs can be valuable resources for patients. Within a limited prompt set, LLMs can generate responses that are accurate, specific, and concise across expert-rated domains. These tools can help bridge gaps in understanding, reinforce provider-patient communication, and empower patients to make informed decisions about their care. However, these tools should not be utilized as a substitute for clinician-provided education. Rather, physician awareness and review of LLM-generated content may be important to understand the information patients may encounter outside the clinical setting.

## References

[1] (2025). The Economist. AI Is Killing the Web. Can Anything Save It?. https://www.economist.com/business/2025/07/14/ai-is-killing-the-web-can-anything-save-it?utm_campaign=shared_article.

[2] Goodman RS, Patrinely JR Jr, Osterman T, Wheless L, Johnson DB (2023). On the cusp: Considering the impact of artificial intelligence language models in healthcare. Med.

[3] Lang SP, Yoseph ET, Gonzalez-Suarez AD (2024). Analyzing large language models' responses to common lumbar spine fusion surgery questions: A comparison between ChatGPT and Bard. Neurospine.

[4] Langford AT, Roberts T, Gupta J, Orellana KT, Loeb S (2020). Impact of the internet on patient-physician communication. Eur Urol Focus.

[5] Bedi S, Liu Y, Orr-Ewing L (2025). Testing and evaluation of health care applications of large language models: A systematic review. JAMA.

[6] Smith AL, Greaves F, Panch T (2023). Hallucination or confabulation? Neuroanatomy as metaphor in large language models. PLOS Digit Health.

[7] Wei Q, Yao Z, Cui Y, Wei B, Jin Z, Xu X (2024). Evaluation of ChatGPT-generated medical responses: A systematic review and meta-analysis. J Biomed Inform.

[8] Stephenson-Moe CA, Behers BJ, Gibons RM (2025). Assessing the quality and readability of patient education materials on chemotherapy cardiotoxicity from artificial intelligence chatbots: An observational cross-sectional study. Medicine (Baltimore).

[9] Sallam M, Snygg J, Allam D, Kassem R, Damani M (2025). Artificial intelligence in clinical medicine: A SWOT analysis of AI progress in diagnostics, therapeutics, and safety. J Innov Med Res.

[10] Aydin S, Karabacak M, Vlachos V, Margetis K (2024). Large language models in patient education: A scoping review of applications in medicine. Front Med (Lausanne).

[11] Yan Z, Liu J, Fan Y (2025). Ability of ChatGPT to replace doctors in patient education: Cross-sectional comparative analysis of inflammatory bowel disease. J Med Internet Res.

[12] Ayoub NF, Lee YJ, Grimm D, Divi V (2024). Head-to-head comparison of ChatGPT versus Google search for medical knowledge acquisition. Otolaryngol Head Neck Surg.

